# Socioeconomic Importance of the Banana Tree (*Musa Spp.*) in the Guinean Highland Savannah Agroforests

**DOI:** 10.1100/2012/350258

**Published:** 2012-04-26

**Authors:** Pierre Marie Mapongmetsem, Bernard Aloys Nkongmeneck, Hamide Gubbuk

**Affiliations:** ^1^Department of Biological Sciences, Faculty of Sciences, University of Ngaoundere, P.O. Box 454, Ngaoundere, Cameroon; ^2^Department of Plant Biology, Faculty of Sciences, University of Yaounde I, P.O. Box 818, Yaounde, Cameroon; ^3^Department of Horticulture, Faculty of Agriculture, Akdeniz University, 07059 Antalya, Turkey

## Abstract

Home gardens are defined as less complex agroforests which look like and function as natural forest ecosystems but are integrated into agricultural management systems located around houses. Investigations were carried out in 187 households. The aim of the study was to identify the different types of banana home gardens existing in the periurban zone of Ngaoundere town. The results showed that the majority of home gardens in the area were very young (less than 15 years old) and very small in size (less than 1 ha). Eleven types of home gardens were found in the periurban area of Ngaoundere town. The different home garden types showed important variations in all their structural characteristics. Two local species of banana are cultivated in the systems, *Musa sinensis* and *Musa paradisiaca*. The total banana production is 3.57 tons per year. The total quantity of banana consumed in the periurban zone was 3.54 tons (93.5%) whereas 1.01 tons were sold in local or urban markets. The main banana producers belonged to home gardens 2, 4, 7, and 9. The quantity of banana offered to relatives was more than what the farmers received from others. Farmers, rely on agroforests because the flow of their products helps them consolidate friendship and conserve biodiversity at the same time.

## 1. Introduction

Agroforestry systems aim to optimize the benefits from biological interaction created where trees and shrubs sometimes are deliberately combined with crops as well as animals [1, 2]. They promote many forms of diversity in the agro ecosystem such as creation of habitats for wild life and beneficial organisms, offering greater diversity of products and lowering the need for external inputs [3–5]. Agroforestry systems have huge potential in meeting the challenges of food security, due to increasing population and degraded environment [6–8]. For a variety of agroforestry systems found in Cameroon, home gardens are among the most favoured land use systems as they enhance the farming family's nutritional and income status considerably [9–11]. Diversification of their production systems has become a keystone to the sustainability of those families. Fruit tree species play an important role in the socioeconomy conditions of the farmers. Bananas are among the most preferred fruit tree species growing in this traditional system but their production remains insufficient for the population. Also, the available species are local; therefore, it is important to evaluate the existing banana production as well as the socioeconomic and ecological characteristics that result from these interactions. As has been pointed out, policies that promote the linkage between domestication and commercialization of nonwood forest products are one of the important areas for further work [12]. In this regard, there is also a need for better integration of food needs and other products with those of the subsistence farmer [13]. Very few works have been done on this problem at the national scale. The first step is the development of better knowledge of the potential utilization of the various species, products, and of the constraints associated with banana production.

 The global aim is to evaluate the structure and the functioning of the GHS agroforests.

The specific objectives are to identify the different types of home gardens existing in the area and evaluate the flow of banana (production, consumption, gift, and commercialization).

 This information will serve as baseline to develop appropriate management techniques. These techniques could help farmers introduce and grow new species of banana in order to get maximum benefits from their production system.

## 2. Materials and Methods

### 2.1. Study Site

The Guinean Highland Savannah (GHS) is located between latitude 7°2′36 N and longitude 13°34′72 E. The climate is guinean type with one active dry season (October–March) and a rainy season covering the remaining of the year. The yearly average total precipitation is 1315.6 mm with a yearly total mean evaporation of 1902.95 mm. The distribution of the rainfall is monomodal. Two main winds blow in the region notably the monsoon during the rainy season from the South and the Harmattan from the North responsible for the drought [11, 14]. The soil of the area is rich in ferruginous compounds derived from granites, granodiorites, and of gneiss after rejuvenation and is composed of red ferralitic developed on old basalts [15]. The vegetation is mainly composed of shrubby and/or woody savannahs with consistent predominance of *Daniellia oliveri* and *Lophira lanceolata* [16]. Nowadays, the density of these species has significantly decreased under the influence of human activities [17].

### 2.2. Methodological Approaches

This study was undertaken in the periurban zone of Ngaoundere notably in Gangassaou, Dene, Sabongari, Wack, Tagboum, Sassa Mbersi, Biskewal and Daran. A total of 187 households distributed in four ethnolinguistic groups (Fulbe, Gbaya, Mboum and Dii) were interviewed and a systemic approach was used. Participatory and reiterative ethnobotanical interviews were conducted through questionnaires containing open, closed and oriented questions. In each household a detailed survey of the composition and management practices were made. The main rubrics of the questionnaire dealt with the main characteristics of farmers (farmer age, gender, family size, beliefs, matrimonial status, strategies used) and agroforests (age, area, inputs), main crops cultivated and/or protected species; constraints.

On the basis of the typology elaborated, the second phase of the work was carried out with farmers verse with technological innovations and who agreed to collaborate with the authors. Only 110 of them accepted to work with the team. Investigations focused only on the banana production. Evaluation in each household included the quantity of banana produced, consumed, commercialised, offered or received from other farmers, the price, incomes generated, and so forth. Farmers involved in the study were train to manage the record book. Home gardens were visited twice a month and discussion held with the garden head.

### 2.3. Data Collection and Analysis

Structural and functional parameters were computed and descriptive statistics were used. For classification of the 110 home gardens, a hierarchical cluster analysis was applied using age, size as main variables. Correlation and regression analyses were used to determine relation between banana production parameters on annual basis. The statistic programs used were Statistica and Statgraphic plus 6.

## 3. Results

### 3.1. Farmer' Socioeconomic Characteristics

#### 3.1.1. Beliefs, Gender and Land Acquisition

Investigations showed that 80% of the respondents are Muslims. 94% of the respondents were men against 6% of women. Regarding the tradition of the area, the land is acquired by donation or inheritance from parents. In each village, the Chief “Djaoro” distributes land. But due to the urbanisation and the extension of the town toward the neighbourhoods, land transactions have been reported at Daran, Biskewal, and Dang. About 55.70% of gardens have been obtained from the chief and 45.63% inherited from parents. However, the farmer can lose his property as soon as he/she leaves the village. In addition, according to the beliefs of Muslims, only men can receive the inheritance from the parents; however, in the other groups like Gbaya and Dii, men as well as women can get it. In these groups, women have their farm different from the one of their husband. In the Foulbe, women are not allowed to work in the farm. They have small plots where they grow some vegetables (*Hibiscus sabdariffa*, *Hibiscus exculenthus*, *Solanum spp.*, *Brassica oleracea, Sesamum indicum, Capsicum fructescens, etc.*).

#### 3.1.2. Farmer Ages

The percentage of the farmers' ages varied significantly (0.0419 < 0.05) from 9.20% who were below 30 years to 28.80% in the group of farmers with more than 60 years old (Table 1). The majority of the farmers interviewed (60.58%) were over forty years old. Farmers in the age group under 30 years were less represented.

Despite the general trend, disparities exist between the villages. In Sabongari, no peasant was older than 60 years, whereas in Tagboum, none were less than 30 years in age. However, in Dene, farmers with ages between 41 and 50 years did not exist.

#### 3.1.3. Matrimonial Status and Scholarship

Almost 62.76% of the farmers were monogamous against the 27.58% who were polygamous (Table 1). Bachelors and widows occupied 6.56 and 5.67%, respectively. About 72% of farmers with age less than 51 years old were monogamous. Despite the fact that the majority of the population were Muslim, young farmers have chosen monogamy. In Sassa Mbersi, there were no bachelors and no widows. There is a significant difference (0.000 < 0.001) among the farmers in terms of marital status.

Concerning the intellectual level of the population, the analysis shows that more than 50% of farmers are illiterate. Among those who have been in school, 30% attained the primary school whereas 19.46% reached the second cycle of secondary school and only 9.12% went up to the level of upper sixth. The highest percentage of literacy was in Mbe. The possible explanation is that Mbe is the Administrative centre of the Mbe Subdivision. In addition, there exist many educational centres. For the other villages where illiteracy is high, it is due to the fact that children have to spend their time looking after cattle instead of going to school. The Dii people are known to be good herdsmen in the Adamawa region.

#### 3.1.4. Family Size

Among the farmers interviewed, 55.56% live with 5 to 10 persons in the homegarden whereas 2.26% live with 21 to 30 persons. The number of persons living in home gardens is significant (0.000 < 0.001). Similar result has been obtained in East Asia [18] by Mapongmetsem et al., in 2000. This general trend with high number of family members (21–30) is peculiar at the village level (Table 1). Illustration is given by Gangassaou, Sabongari, and Mbe villages. However Sassa Mbersi is singularised by the absence of families with more than 15 persons. Despite the high number of people under the responsibility of the head of the family, there is no significant correlation between the family size and the active member who can help the head of the family during farming works (*r* = 0.081; 1.564 > 0.05). Chiefs of family in their majority (46.6%) have 2 to 3 active persons.

 For farming activities, 92% of the farmers do not receive help from visiting family members who spend some of their times in the household. Small fractions among them (8%) receive help from 1 to 5 persons. Fortunately, collective help exists in each village of the region locally known as “surge” to solve the problem of labour. There is no significant correlation (*r* = 0.2; 0.8913 > 0.05) between farmer age and family size nor between active members considered (*r* = 0.046; 1,234 > 0.05).

### 3.2. Agroforest's Characteristics

#### 3.2.1. Home Gardens Age

The distribution of home gardens per village and age shows that 35.3% of home gardens are less than 15 years old. Home gardens of 15 to 30 years are ranked second (28.45%), those of more than 45 years, third (19.19%), and lastly those of 31–45 years (16.97%) (Table 2). This result is comparable with that obtained in East Asia [18]. In some ethnolinguistic groups like the Dii, home gardens farming has been recently introduced.

 Home gardens more than 30 years old are absent in Sabongari as compared to those which are 15–30 years old. The situation is due to the fact that this village has been created recently between 1970 and 1980 with the massive migration of the Dii population from the Savannah to near the road [19, 20]. There is no significant correlation between the farmer age and that of home gardens (*r* = 0.4; 0.823 > 0.05).

#### 3.2.2. Home Gardens Area

Three landholding sizes were identified: small (<1 ha), medium (1-2 ha), and large (>2 ha). Similar results have been reported in Kerala [21]. Majority (80.37%) of the Periurban agroforests are concentrated in areas less than one hectare (<1 ha) against 8.68% for those in which the surface area is equal to or more than 2.1 hectares (Table 2). In the rural zone, the same trend is observed. The explanation is given by the traditional law which limits the area per inhabitant [22]. Exceptionally farmer can go beyond 0.25 ha. In Sabongari, 15.4% of home gardens with more than 3 ha are found (Table 2). Some of them are located at the beginning whereas others are situated at the end of the villages along the main road.

This information is very important in the elaboration of the typology of home gardens of the region which could serve as baseline to address efficiently the various constraints enumerated by farmers in the region.

#### 3.2.3. Differentiation Multistrata Type

**Figure 1:**
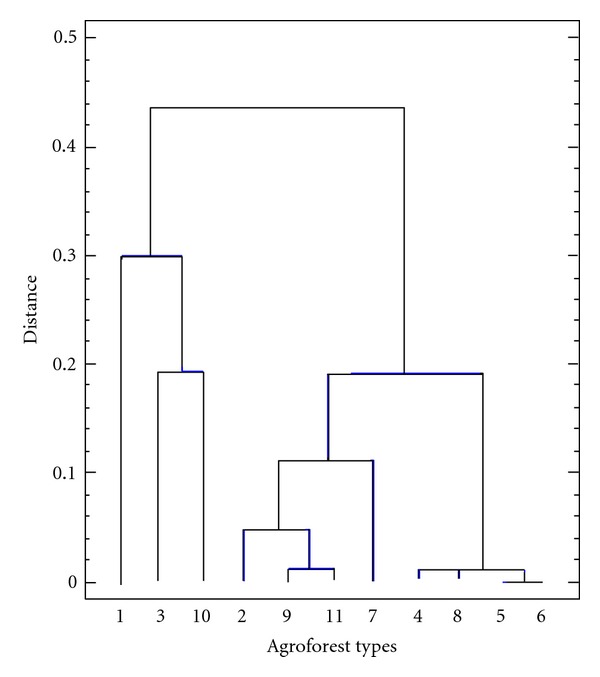
Hierarchical classification showing 11 types of agroforests existing in the tropical Guinean Highland savannahs region.

The garden's ages as well as their surface areas are the two important criteria to distinguish the home gardens. On the basis of a cluster analysis using the nearest neighbour method and the squared euclidean, the 187 selected households were categorised into 11 types (Figure 1). These different types of agroforests are conceivedand managed by farmers, over the Guinean Highland Savannahs (GHSs).

#### 3.2.4. Structural Characteristics

Every house has fruit trees growing, although not all are productive. Less than a quarter of all fruit trees recorded actually bear fruit. Those without fruits were newly planted and are expected to start giving fruit within the next few years. Once these trees start producing, the potential value of the crop per household and for the whole village will be substantially higher.

The Guinean Highland Savannah agroforests harbour a substantial diversity of tree species. The number of species in the various traditional system types ranges from 23 to 62 species. The maximum number of layers found generally in home gardens is 5 [23]. Five stratas are effectively present in Types 5, 9, 10, and 11 which are old whereas only four of them exist in the rest. The layer number three is absent in Type 7.

Concerning the age criteria, home garden Types 1, 2, and 3 are young (less than 15 years) whereas 9, 10, and 11 are the oldest (more than 45 years old). The rest of types are intermediate.

For the area, home garden Types 1, 3, 6, and 9 are very small (less than 1 ha) whereas Types 5, 8, and 11 are the largest (more than 2 ha). Types 2, 4, 7, and 10 are intermediate (1-2 ha). The most predominant agroforestry home garden types in the GHS are 1 (26.174%), 3 (22.148%), 6 (16,107%), and 9 (20.142%) types. Similar characteristics are reported in tropical Asia mainly in India [21].

Here in after are described the main traits of the eleven types of homesteads found in the GHS.

**Type 1**

Gardens belonging to the Type 1 are the most represented (26.17%). It is found in all the villages of the region. In Sassa Mbersi and Dene, 15.38% of the owners do not have other farms. The majority of farmers belonging to this group are between 30 and 50 years old and 72% of them are monogamous. They practice cattle farming (12%) whereas 46% of them rear goats and sheep. A total of 16 species are present among which are 9 crops and 7 fruit trees.

**Type 2**

It represents 5.36% of the study sample. They are found in Gangassao, Dene, Sasa-Mbersi, Wack, and Mbe. Young (<30) and old farmers (>60 years) are absent. Of these 50% are cattle farming farmers. A total of 19 species are found among which 11 are crops.

**Type 3**

This type represents 22.81% of the total sample and is found in all the villages. It is more abundant in Sabongari. Cattle percentage is 24% against 40% for goats and sheep. The total number of plant species found is 26 distributed into 13 crops and 13 fruit trees.

**Type 4**

It is less represented (1.34%) and exists only in Sabongari. Their owners have not been to school. Their family size is between 5 and 10 persons. A total of 62 plant species are cultivated. The majority of these species are represented by 46 fruit trees. This type tends to become a forest. The three types of species that are trees of the past, trees of future, and trees of the present described by Limier (1978) are well represented.

**Type 5**

It is concentrated in Wack and Sabongari where it is very abundant (23.07%). All the farmers have more than 30 years old. They rear goats and cattle. The number of plant species is 47 among which are 34 fruit trees like in Type 4.

**Type 6**

This type represents 16.81% of the sample. It is found in 5 villages among which Gangassaou, Daran, Biskewal, and Wack (29.62%). Some of its owners (8.33%) do not have another type of farms. About 23 plant species are cultivated there among which 10 crops.

**Type 7**

This type representing only 2.01% of the sample is found in Gangassaou, Dene, and Wack. Its owners in majority (66.66%) are more than 60 years old. This result suggests that the three villages are ageing. Among these farmers, 33.33% do not have another type of farms. In addition they are monogamous. They rear only chickens. The number of plant species is 36 with 15 crops and 21 fruit trees.

**Type 8**

A few farmers (1.34%) are in practice. Farming exists only in Gangassaou and Dene. They are illiterate and rear cattle. Plant species cultivated are composed of 13 crops and 4 fruit trees.

**Type 9**

It is one of the most represented types (20.13%) and rank third after Types 1 and 3. It is found in all the villages of the region. Their owners rear various types of animals of whom 13% rear cattle and 30% rear goats and/or sheep. The total number of species found is 25 among which 9 crops and 15 fruit trees.

**Type 10**

It is less represented in the region (1.34%) and is found in Gangassaou and Mbe. Their owners are monogamous and their family size ranges from 16 to 20 persons. They are literate. Among them, 50% rear cattle. They grow 11 crops and 15 fruit trees.

**Type 11**

This type is found only in wack village. It is very old (60 years old) and very large (8 ha). Bush fallowing and rotation are practiced. Some important wild tree species are protected in this type. Among these species are *Borassus Aethiopum, Vitex Spp.*, *Parkia Biglobosa, and vitellaria paradoxa. *these wild edible plants are among the most preferred species, “the top 14” in the area [24, 25]. The number of plant species is high and mainly based on fruit trees.

### 3.3. Functional Characteristics

**Figure 2:**
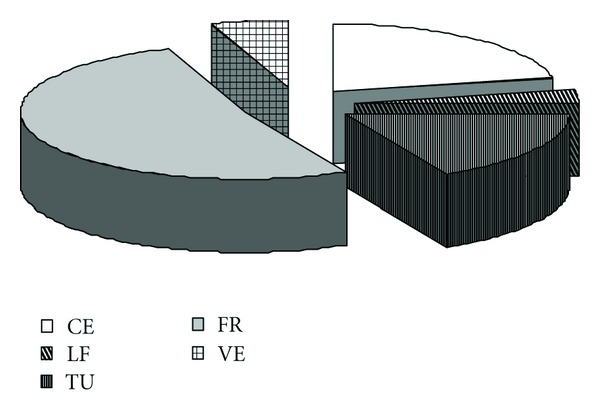
Diversity of food products in home gardens: cereals (CE), tubers (TU), leguminous food (LF), fruits (FR), and vegetables (VE)

The eleven types differ in their functional characteristics. Various crops are grown in tropical multilayer agroforestry systems. Due to the nutrition habits and choice of the population, the crops are grouped into main categories such as cereals, tubers, leguminous foods, vegetables, fruits, wood, and medicines. Fruits, tubers, and cereals are the most important in the region (Figure 2). The quasitotality of the production in home gardens is consumed. However, the surplus of products could be sold in order to buy products which are not produced by the farmers. The incomes generated from the commercialization of the various products help to buy soap, meat, salt, oil, clothes, and so forth. The management of the system varies according to the socioeconomic strategy of the head of the family. Two main choices that are commonly practised in the region are subsistence and marketing strategies. The fruits are the most important products sold directly by the farmers along the road or in the local and/or regional markets. The most common fruit tree species represented in the system are *Persea americana *(87.75%), *Citrus limon *(75.79%), *Mangifera indica *(73.56%), *Musa spp.* (73.52%), *Anacardium occidentale* (68.58%), and *Carica papaya *(69.07%). Among these species, bananas occupy an important rank and contribute equally to income generation in the family while contributing to the daily diet of the family. It appears necessary to estimate its production in the context of the GHS of Cameroon.

 The total amount of food produced in gardens evaluated is equal to 23.195 tons among which fruits (11.99 t), cereals (5.07 t), and tubers (4.01 t) contribute the most (Figure 2). These foods are mostly consumed. According to the farmer's choices and strategies, the excess products can be commercialized in order to buy other products and/or to pay school fees of children.

The contribution of the fruits on the gardens production is consistent. Bananas are among the main fruits consumed in the region but only a few species grow in the Guinean highland savannahs. The quantity produced is not enough to supply the population. In addition, only local species are grown by farmers. This situation induces farmers to import considerable quantities of banana and plantain from the humid lowland forest of Cameroon. Thus, it is necessary to appreciate its production in this tradition system in order to estimate the approximate quantity for importation as well as to develop strategies to introduce new banana species. The different types of gardens can be classified along a gradient from small to big banana producers.

### 3.4. Banana in the System

#### 3.4.1. Description of Banana Tree

Two local species of banana are cultivated in the region. They are *M. sinensis *var. *petit nain* and *M. paradisiaca* locally known as kouni and kodon in Fulfulde, respectively. The tree of the first species is short (2.5 m to 3 m). *M. paradisiaca* is very tall (6 to 8.5 m). The bunch size of each species as well as the number of hands and fingers varies according to the management techniques. Also, fruits of *Musa paradisiaca* are hard like what is found in plantain. Maybe, it is why they call it locally “kodon” which means plantain. Banana is consumed and commercialized. However, the size remains small as compared to the improved species and varieties grown in the Humid Lowland forest. When ripped, bananas are eaten and cooked when unripe depending on the ethnolinguistic habits of the group.

#### 3.4.2. Management of Banana Tree and Uses

There is no special management of the banana tree in this system concerning the fertilisation. Farmers do not used chemicals as it is usual in the multistorey agroforestry systems of the area. Organic matter is the main input used by the farmers. The soil fertility is maintained through dust, animal dungs, and litter from trees toward decomposition. The propagation of the tree is by suckers.

 Very often, the farmers remove old and dried leaves of the banana tree and put them under the tree. They cut young leaves for domestic uses (conservation of food or meat, etc.). Different parts of the tree are also used in traditional medicine. Yellow leaves associated with other plants (*Bidens pilosa*, *Psydium guajava*, *Harungana madagascariensis*, *Chrisanthellum americanum*, etc.) are decocted to cure typhoid fever whereas those associated with egg and fried treat diarrhea and amoeba. Bananas have been integrated in this traditional system as a vital component of the local diet and economy. In areas where erosion is common, farmers use *Musa sinensis* to stabilize the soils (to control erosion).

#### 3.4.3. Yield of Banana and Significant Relationship between Farmers

**Figure 3:**
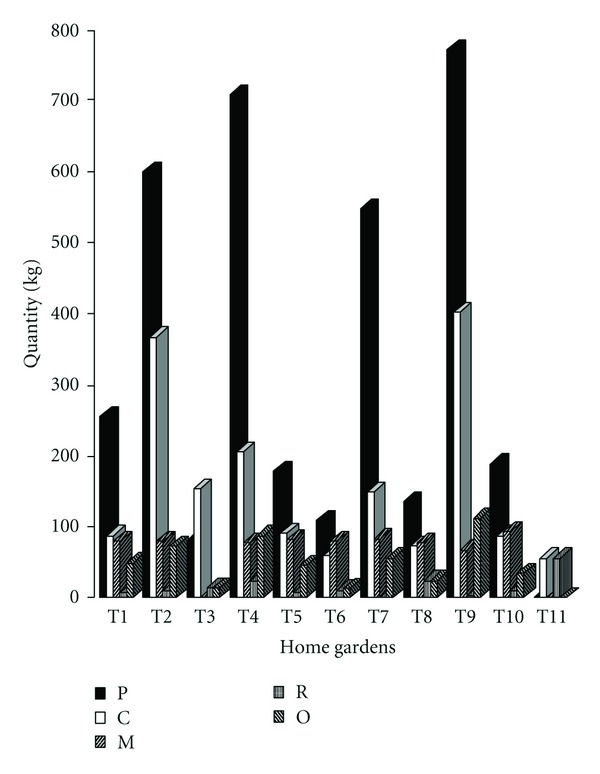
Banana annual flow in 11 agroforests (Type 1, 2,…, 11): production (P), consumption (C), marketing (M), reception (R), and offers (O).

The banana production varies from farmer to farmer depending on the importance given to the product. The total mean yield of banana in the periurban home gardens is estimated at 3.57 tons. Year^−1^. Three categories of banana producers exist in the area. The first group for which the quantity produced is more than 300 kg, is belonging to agroforest Types 2 (598 kg), 4 (707 kg), 7 (546 kg), and 9 (770 kg) types. The second group comprises those who produce between 100 and 300 kg that are represented by gardens of Types 1, 5, 6, and 10 whereas the last category regroups those of 3 and 11 types which produce less than 100 kg of banana per year (Figure 3). Significant differences exist between the different types of homesteads found in the area.

 The total quantity of banana consumed in the periurban zone is 3.5439 tons year^−1^ (93.5%). The quantity consumed can derive from the farmer's own production or from gifts by relatives. Farmers who do not grow banana receive them free from their neighbours. It is the case of those belonging to the eleventh type of home gardens (Figure 3). Part of the periurban production is sent to the urban zone as gift or sold for food.

Tropical agroforest plays an important social role beyond the economic and ecological aspects by promoting the family integration through sharing farming, managing, harvesting activities, and products with other members of the communities. In this regard, the total quantity of banana offered to relatives is 0.55 tons against 0.112 tons received. Among the household evaluated, gardens belonging to Type 11 do not cultivate banana. Nevertheless, it can be mentioned that, in rare cases, the two species are found in the same plot. In general, there is a tendency for the supply to exceed demand in all the garden types except for Type 11 where the quantity of banana supply is equal to demand (Figure 3). There is a significant correlation between banana production and consumption (*r* = 0.834; 0.00013 < 0.001). Generally, the banana produced in agroforests is consumed (93.5%). Only a few quantities (6.5%) are commercialized.

### 3.5. Commercialisation of Banana

The species are commercialized either ripe or unripe. It is sold in bunches, hands, and/or fingers. When the fruit reaches the consumption maturity state, the cost of one is 10 francs CFA. The bunch costs 600–1200 francs CFA depending on its size. Due to the fact that plantains are difficult to grow in the area, *Musa paradisiaca* is more preferred by the local population. The total quantity sold in local markets is 1.01 t. Very often, “buyam and sellam” buy banana and plantain from the humid lowland forest and distribute them all over the two agro-ecological zones (Guinean highland savannahs, Sudano-sahelian). In some cases, these products are transferred to neighbouring countries markets (Chad, Central African Republic). Despite the fact that each farmer belongs to a particular type and susceptible to sell agricultural products, the quantity sold varies among the farmers. Farmers from the agroforests Types 2, 4, 7, and 9 are the main sellers. The mean annual income generated is 1689.14$. The income generated helps the farmers to pay school fees of children, food that they do not produce, oil, salt, meat, and animals, and manufactured products.

## 4. Discussion

The majority of the farmers interviewed were Muslims. The high percentage of the above group can be explained by the double domination of the Adamawa population by Ousman Dan Fodio and Lamido of Rey-Bouba [19, 20]. Existence of the Christians stems from the fact that they had contact with the Germans. Also, the installation of the colonial administration in the Wack's village favoured the contact during this period. The rest of ethnolinguistic groups were still dominated by the Lamido of Ngaoundere [19]. This result suggests that home gardens are owned by men. Women who are head of family are rare. They are represented by widows or bachelors. Widows inherited farm from their late husbands whereas bachelors can obtain them from their parents either while they are alive or after the death. Among the Dii ethnolinguistic group, men as well as women can inherit the “properties” from their parents.

Their education level is low. The representation of the women is low. Predominance of monogamy in the periurban zone of Ngaoundere is in disagreement with the polygamy found Muslims areas [25]. The majority of agroforests found in the zone are very young and their area is less than one hectare. This can be due to the recent installation of the population. It can be also due to the mobility of the population. Similar results were reported in the Sodano-Guinean savannahs [26]. Eleven home garden types exist in the Sudano-Guinean savannah of Cameroon. They showed important variations in all their structural characteristics.

The results of this work indicate that a considerable array of plant species are actively cultivated in this traditional agroforestry system in GHS on residential plots and that they have a significant cash and home consumption value. The production of the system is usually consumed; only small quantities are sold. In the South African rural villages, similar trends have been observed [27]. The preponderant use of garden products for domestic consumption indicates that from the point of view of food security, it is a valuable option for small-scale farmers [28]. These findings corroborate those of other studies also indicating the importance of fruits to rural communities [22, 27, 29, 30].

The contribution of the banana production to the agroforest's yield is consistent. Among the 11 types found, the main banana producers belong to home garden Types 2, 4, 7, and 9. However, the banana production in the GHS agroforests is lower than that obtained in agroforests in the humid lowland forest zone of Cameroon [29]. The major constraints for banana are climate and soils. The quantity produced remains insufficient to satisfy local demand. It has been beneficial for the population to introduce new varieties in the area. The study demonstrates the important socioeconomical role played by tropical home gardens. The diversity of species found in the Guinean Highland Savannahs' agroforests may serve as another example for the suggestion of Janzen (1998) by Khaya et al. [31] for which “A gardenification of the tropics is capable of preserving large lumps of wild biodiversity”. The flow of products in the system is important in consolidating friendship relation among the farmers.

Thus, the growing of crops for home consumption represents considerable cash saving. This work complements others demonstrating the multifaceted nature of rural livelihoods. Coupled with the extraction of resources from adjacent communal lands it indicates the importance of small-scale activities and secondary resources in contributing to food security, household well-being, and the informal economy.

Besides being a potentially interesting source of income, banana also contributes to the structural diversification of the agroforests, which is important for creating habitat for local fauna and flora. Farmers rely on agroforests for their food, income, creation, and consolidation of relationships, treating diseases and reducing spending money.

## Figures and Tables

**Table 1:** Farmer percentages according to their socioeconomic characteristics (age, marital status, family size) in nine villages of the Guinean Highland Savannahs. BA; Bachelor, MO; Monogamy, PO; Polygamy, WI; Widow.

Villages	Age (years)					Marital status				Family size				
	<30	30–40	41–50	51–60	>60	BA	MO	PO	WI	<5	6–10	11–15	16–20	21–30
Gangassaao	7,9	27.7	28.9	18.4	21.0	7.9	60.5	31.6	0	13.1	52.6	15.8	15.8	2.6
Sabongarii	7.7	23.1	23.1	7.7	28	7.7	46.1	46.1	0	15.4	61.5	15.4	0	7.7
Dene	7.7	46.1	0	23.1	23.1	30.8	30.8	20.1	15.4	46.1	38.5	23.1	7.7	0
Tagboum	0	20	28	24	26.7	4	76	16	4	20	72	4	4	0
Sassa Mbersi	7.7	23.1	23.1	7.7	28	0	84.6	15.4	0	7.7	84.6	7.7	0	0
Wack	3.7	25.9	14.8	18.5	37	3.7	70.4	22.2	3.7	14.8	59.2	18.5	7.4	0
Mbe	5.7	31.0	18.9	22.5	20.1	5	60	15	20	15	40	30	5	10
Biskewal	35	5.8	15	16.7	28.5	8.8	61.2	23.8	6.33	21	59	14.5	5.5	0
Daran	7.5	25.5	0	24	43	25.5	70	3.5	1	14.5	72	7.5	6	0
Mean	9.2	25.5	16.9	17.0	28.8	6.56	62.8	27.6	5.7	16.6	55.6	5.6	5.6	2.3

**Table 2:** Distribution percentages of home gardens according to age and area in villages.

Villages	AGE (years)				AREA (ha)			
	<15	15–30	31–45	>45	<1	1–2	2.1–3	>3
Gangassao	21	31.60	18.40	28.90	86.8	10.5	2.7	0
Sabongari	15.4	84.60	0	0	61.5	15.4	7.7	14.4
Dene	53.8	23.10	15.4	7.7	76.9	8.5	11.5	3.1
Tagboum	36.0	16.00	24.00	24.00	80	4	12	4
Sassa Mbersi	61.5	15.4	7.7	7.7	84.6	15.4	0	0
Wack	14.8	18.5	33.33	33.33	85.2	11.1	0	3.70
Mbe	45	10	20	25	85	15	0	0
Biskewal	56	4	30	10	76.5	5.5	10	8
Daran	48	24	17	21	87	10.5	2.5	0
Mean	39.06	25.24	18.43	17.51	80.39	10.70	5.16	3.69
